# Central Integration of Canal and Otolith Signals is Abnormal in Vestibular Migraine

**DOI:** 10.3389/fneur.2014.00233

**Published:** 2014-11-10

**Authors:** Susan King, Joanne Wang, Adrian J. Priesol, Richard F. Lewis

**Affiliations:** ^1^Boston University, Boston, MA, USA; ^2^Jenks Vestibular Physiology Laboratory, Massachusetts Eye and Ear Infirmary, Boston, MA, USA; ^3^Brown University Medical School, Providence, RI, USA; ^4^Department of Otology and Laryngology, Harvard Medical School, Boston, MA, USA; ^5^Department of Neurology, Harvard Medical School, Boston, MA, USA

**Keywords:** vestibular, migraine, cerebellum, perception, oculomotor

## Abstract

Vestibular migraine (VM), a common cause of vestibular symptoms within the general population, is a disabling and poorly understood form of dizziness. We sought to examine the underlying pathophysiology of VM with three studies, which involved the central synthesis of canal and otolith cues, and present preliminary results from each of these studies: (1) VM patients appear to have reduced motion perception thresholds when canal and otolith signals are modulated in a co-planar manner during roll tilt; (2) percepts of roll tilt appear to develop more slowly in VM patients than in control groups during a centrifugation paradigm that presents conflicting, orthogonal canal and otolith cues; and (3) eye movement responses appear to be different in VM patients when studied with a post-rotational tilt paradigm, which also presents a canal–otolith conflict, as the shift of the eye’s rotational axis was larger in VM and the relationship between the axis shift and tilt suppression of the vestibulo-ocular reflex differed in VM patients relative to control groups. Based on these preliminary perceptual and eye movement results obtained with three different motion paradigms, we present a hypothesis that the integration of canal and otolith signals by the brain is abnormal in VM and that this abnormality could be cerebellar in origin. We provide potential mechanisms that could underlie these observations, and speculate that one of more of these mechanisms contributes to the vestibular symptoms and motion intolerance that are characteristic of the VM syndrome.

## Introduction

Several characteristics of the symptoms and signs associated with vestibular migraine [VM, Ref. (1)] led us to consider the possibility that a component of the underlying pathophysiology could relate to abnormalities in the brain’s synthesis of inputs from the semicircular canals and the otolith organs. The former sense angular head velocity, the latter the vector sum of gravity and linear acceleration, and the central synthesis of these signals contribute to brain’s estimate of head orientation and motion. We noted from previous work that vestibular symptoms in VM are often provoked or exacerbated by changing head orientation relative to gravity (2); many VM patients develop positional nystagmus during attacks of vertigo (3); and motion sickness sensitivity is more pronounced in migraine patients with vestibular symptoms than in those lacking these symptoms (4). Furthermore, although most of the eye movement features observed in patients with VM are indistinguishable from those seen in migraine patients without vestibular symptoms [(5), whom we will term “migraine controls” or MC], a large study suggested that the time constant of the angular vestibulo-ocular reflex (VOR) was slightly longer in VM subjects and was slightly more strongly suppressed by post-rotational tilt than in MC subjects or normal (N) controls (4).

One consistent underlying feature of these observations is the possible contribution of abnormal canal–otolith integration by the brain. In particular, changing head orientation relative to gravity (e.g., turning in bed) modulates activity in canal and otolith afferents simultaneously, so abnormal symptoms and signs in this situation suggests a possible deficit in the canal–otolith integrative process; motion sickness is attributed to sensory conflict (6), but this conflict can be between senses (e.g., vision and vestibular) or within a sense (between the canal and otolith signals generated by passive head motion); and finally the dynamics of the VOR and the effect of head tilt on the VOR’s duration depend on suppression of activity in the vestibular nuclei by projections from the cerebellar nodulus and uvula, the brain region that synthesizes canal and otolith signals (7).

As noted above, while a variety of eye movement abnormalities have been described in patients with VM, many of these studies have been marred by the use of normal people as control subjects rather than MC subjects. Indeed, when VM patients are compared to MC subjects, these eye movement changes are almost always identical in both groups (5), indicating that they are associated with migraine but not the presence or absence of vestibular symptomatology. Since recent work has suggested that vestibular-mediated eye movements and percepts are generated by divergent pathways and mechanisms in the brain (8, 9), with the former more dependent on the frequency of head motion and the latter more reflective of canal–otolith integration in the brain, we proposed that by primarily focusing on perception we could potentially uncover abnormalities that were specific to VM.

We have addressed this issue with several experimental approaches. These studies are still in progress and the results that we present below are preliminary. Since definitive conclusions cannot yet be drawn from this work, we are presenting our findings in the framework of a “hypothesis and theory” format. Below, we present our preliminary results from these studies, each of which used motion paradigms that concurrently modulated activity in canal and otolith afferents. In the first study, we measured vestibular *perceptual thresholds* during *roll tilt* about an earth-horizontal axis, which provided the brain with a *co-planar* canal and otolith signal; the second study focused on measuring *eye movements* and *perceptual tilt estimates* during fixed-axis *centrifugation*, which provided a *conflicting* canal (yaw) and otolith (roll) motion cues; and finally we measured *eye movements* during a *post-rotational tilt* paradigm that also provided *conflicting* canal and otolith cues. As discussed below, we believe that the results of these three studies are consistent with abnormal synthesis of canal and otolith cues by the brain, and based on these results, we offer hypotheses about potential mechanisms that could explain some of clinical features of VM.

## Methods/Results

### Patient selection

For each of the three experiments, we tested three groups of subjects – VM, migraine controls, and normal controls. VM subjects were defined using “Neuhauser” criteria for definite VM (1), which requires repeated episodes of vestibular symptomatology, repeated headaches that meet International Headache Society (IHS) criteria for migraine, a temporal relationship between the vestibular and migraine symptoms, and a thorough work-up to exclude other potential otologic or neurologic etiologies. This work-up included a clinical evaluation by an otoneurologist (RFL), normal brain MRI and audiogram, and normal vestibular tests that include bi-thermal calorics and earth-vertical rotational testing. Migraine controls had histories of headaches that met IHS criteria for migraine, no history of otologic or other neurologic disease, no history of vestibular symptoms of any type, a normal exam by the otoneurologist, and normal audiogram, brain MRIs, and vestibular testing. Normal controls had no history of otologic or neurologic problems (and specifically no history of headache or dizziness), normal exams by the otoneurologist, and normal audiograms and rotational tests. We included migraine patients who experienced non-vestibular auras, but excluded subjects with chronic migraine.

### Experiment 1: Roll tilt perceptual thresholds

Migraine can be conceptualized as a disorder of sensitization (10), so one way to assess its effect on the vestibular system is to measure perceptual thresholds, the magnitude of head motion where the brain can first correctly perceive the presence or direction of motion (11). We previously measured perceptual thresholds using a direction-discrimination task for VM, MC, and N subjects who were studied during roll tilt about an earth-horizontal axis (co-planar modulation of canal and otolith cues), roll rotation about an earth-vertical axis (canal-only stimulation), and very slow “quasi-static” roll tilt [otolith-only stimulation; (12, 13)]. The two dynamic protocols (roll tilt, roll rotation) were limited to two frequencies (0.1 Hz, 1.0 Hz) because of time constraints due to the lengthy testing procedure, and we found that roll tilt thresholds were lower in VM subjects at 0.1 Hz but not 1.0 Hz, and that all three groups were equivalent when tested with roll rotation and quasi-static roll tilt. Based on these results, we proposed that the motion signal in the brain was enhanced in VM patients when canal and otolith cues were both provided, which was evident at 0.1 Hz but not 1.0 Hz because the canal cues dominated the response at the higher frequency. There were several potential problems with this study: otolith cues were not studied dynamically, as could be done using linear translation at different frequencies, so the possibility remains that the roll tilt change in VM was actually due to increased dynamic sensitivity of the otolith organs; tactile cues were minimized but the sensitivity of the skin to touch and pressure was not quantified (although visual and auditory cues were removed or minimized, as tests were performed in the dark and subjects wore headphones that provided white noise); and perhaps most importantly, we found this threshold change only at one of two frequencies, as time constraints did not allow us to test thresholds at other mid-frequencies near 0.1 Hz, where similar changes would be expected if the results indeed reflected an enhanced sensitivity to motion driven by abnormal canal–otolith integration.

**Figure 1:**
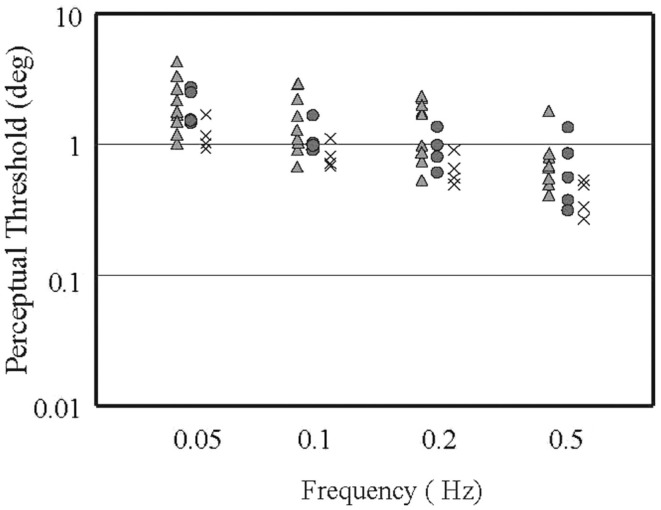
**Roll tilt perceptual thresholds (in degrees) versus frequency of the sinusoidal movement [see in Ref. (15) for methodological details], for normal subjects (triangles), migraine subjects with no vestibular symptoms (circles), and subjects with vestibular migraine (crosses)**. Each icon represents the threshold measure in a given subject at a given frequency. The icons are offset horizontally for clarity. Note that both axes are logarithmic.

We have begun a more definitive study, therefore, which specifically addresses each of these potential shortcomings. In our prior study and in work done in normal subjects, it is clear that at very low frequencies (where the canals are relatively insensitive to head rotation) thresholds are defined by the otoliths, and at frequencies at and above 1.0 Hz, the canals are so much more sensitive than the otoliths that thresholds are defined by the canals (12, 14). We therefore sought to study multiple frequencies between these end-points, where canal and otolith sensitivities are roughly the same order of magnitude and where non-linear integration of these two vestibular cues should therefore be most evident. To date, we have studied Four VM subjects, five MC subjects, and Ten normal controls (not all subjects have been tested on all frequencies yet). As shown in Figure 1, when we examined VM, MC, and N subjects using our roll tilt perceptual threshold method at frequencies ranging from 0.05 to 0.5 Hz, we found that thresholds in N subjects (triangles) were distributed over a fairly wide range, MC thresholds (circles) fell largely in the central region of this range, but VM subjects had thresholds (crosses) that were clustered at the lower end of the normal range or were below the smallest normal threshold. Overall, the mean threshold did not differ between the normal subjects and MC subjects (*p* = 0.24) but did differ significantly between the VM subjects and the normal (*p* = 0.02) and the MC subjects (*p* = 0.04). While we must complete this study by testing more VM and MC subjects, perform the control (roll rotation, inter-aural translation) studies, and quantify tactile sensitivity using Semmes–Weinstein filaments (15), these preliminary results provide preliminary conformation of our prior results, namely that perceptual thresholds during roll tilt at mid-frequencies are lower in VM than control groups. This finding suggests that by presenting co-planar roll canal and roll otolith signals simultaneously to the brain, the central estimate of motion is somehow enhanced in the VM population.

### Experiment 2: Roll tilt perception and eye movements during centrifugation

Experiment 2 differs from the roll tilt perceptual threshold experiment in several ways: we measured perceptual estimates of the amplitude of head tilt (e.g., “magnitude estimation”) rather than perceptual thresholds; we measured eye movement responses simultaneously; and the motion paradigm provided conflicting (orthogonal) rather than co-planar canal and otolith cues. The motion paradigm involved rotating subjects in yaw about an earth-vertical axis (accelerating from 0 to 200°/s over 15 s, holding angular velocity constant for 120 s, and then symmetrically decelerating to a stop) with the subject displaced laterally from the rotational axis along the inter-aural plane and facing the direction of motion. This paradigm therefore presents, in addition to the yaw canal cue, an inter-aural centrifugal force, which rotates the gravito-inertial force (GIF) sensed by the otolith organs by 20° about the roll axis (16). The key difference between this paradigm and the roll tilt experiment is therefore the conflict, rather than concordance, between the yaw canal signal and the roll otolith signal. Subjects provided an estimate of their perceived tilt by orienting a “somatosensory” bar (17), which was grasped at each end, so they perceived it to be parallel to ground (e.g., perpendicular to gravity), and two-dimensional (horizontal, vertical) eye movements were measured simultaneously using a head-mounted video system.

**Figure 2:**
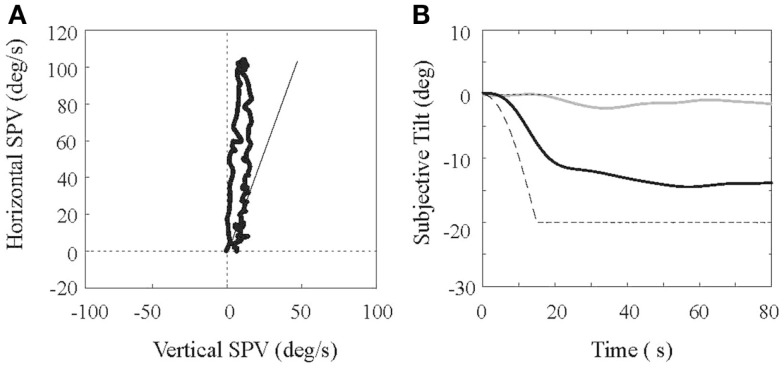
**Eye movement and perceptual responses in a subject during fixed-radius centrifugation are shown**. **(A)** shows the slow phase eye velocity (SPV) with the horizontal velocity on the *y*-axis and the vertical velocity on the *x*-axis. The line represents the orientation of the earth-vertical relative to the head. **(B)** shows measures of perceived tilt, acquired with the somatosensory bar method, during centrifugation. The dashed line shows the tilt of the gravito-inertial force in the roll plane versus time, the light line shows the perceptual response when the head is centered at the rotational axis, and the dark line shows the perceptual response when the head is displaced eccentrically.

An example of results from a normal subject is shown in Figure 2. Eye movements (Figure 2A) are plotted in polar coordinates, with horizontal slow phase eye velocity (SPV) on the *y*-axis and vertical SPV on the *x*-axis. As previously demonstrated in non-human and human primates [e.g., Ref. (18)], the initial VOR response is horizontal, but a vertical component builds up that shifts the eye’s rotational axis toward alignment with the GIF (shown by the solid bar in Figure 2A). The eye velocity then decays toward the origin along a line that approximates the GIF. Figure 2B shows an example of tilt perception when the subject is rotated while centered (gray line) and while eccentric (black line), using the somatosensory task. When centered, minimal tilt is perceived, but when eccentric, a percept of roll tilt develops gradually and slowly approaches the tilt of the GIF (dashed line). This lag between the GIF tilt and the percept of tilt is a well-described phenomenon [e.g., Ref. (16)], and has been interpreted as a strategy used by the brain to avoid the sensory conflict, which would occur if the estimate of gravity shifted away from the earth-vertical while the yaw rotational cue remained strong. Our preliminary data compared eye movement and perceptual results in a small population of VM, MC, and N subjects (6, 5, and 5, respectively), and we found that most measured parameters were equivalent in the three groups. Specifically, the gain and time constant of the horizontal VOR, the size of the eyes axis shift, and the rate of the axis shift, did not differ between groups (*p* > 0.05 for all comparisons). Perceptual responses did differ in the VM subjects, however, as they developed percepts of roll tilt more slowly than the MC and N groups (the tilt time constant for the VM subjects was about twice as long as the control groups; *p* = 0.03 compared to MC and *p* = 0.01 compared to normal subjects), although the ultimate size of the tilt percept was the same in the three groups. Therefore, of all measured parameters, only the dynamics of tilt perception differed between groups. These preliminary results are interesting for two reasons: (a) since the canal and otolith cues are in conflict with this motion paradigm, if their interaction was somehow enhanced in the VM subjects one would predict that their percepts of tilt would develop more slowly; and (b) as alluded to earlier, there is evidence that perception is more dependent on canal–otolith interactions than eye movements (8, 9), and the discrepancy between the dynamics of the shift in the eyes rotational axis (the estimate of gravity accessed by the oculomotor system) and tilt perception (the estimate of gravity accessed by the perceptual system) appears to support this contention. One interesting corollary to these findings is the recent fMRI study, which showed that when the vestibular system was activated with caloric stimulation, that VM subjects differed from MC and normal subjects only in the thalamus (19), a brain region, which would be predicted to influence vestibular-mediated percepts but not eye movements.

### Experiment 3: Eye movement responses during post-rotational tilt

Post-rotational tilt is another standard experimental motion paradigm that investigates how the brain synthesizes rotational and otolith information [e.g., Ref. (20)]. We have performed this experiment on small number of VM, MC, and normal controls to date (6, 4, and 5 subjects, respectively), using standard methods. Specifically, subjects were rotated in yaw about an earth-vertical axis at a constant velocity for 120 s, which allowed the VOR to attenuate, then they were rapidly decelerated to a stop (producing the large reversal of the angular VOR characteristic of post-rotatory nystagmus), and then the head was quickly tilted off the vertical axis (we tilted the head in roll by 45°). This combination of motions presents the brain with a large head-centered yaw angular velocity signal, even though the head is actually stationary, and this is present while the head is tilted off the vertical axis. If the head were actually rotating about its yaw axis, the otolith organs would sense modulation in the orientation of gravity, so the intra-vestibular sensory conflict is driven by the absence of otolith modulation. This type of sensory conflict appears to be resolved by the brain in two complementary ways: (a) the angular velocity signal in the brain is suppressed, evidenced by a rapid attenuation of the horizontal VOR; if the brain does not sense the yaw rotation then the conflict is resolved; and (b) the brain shifts the estimate of head angular velocity axis so that it is no longer aligned with the head’s yaw axis but rather is aligned with gravity. If the head is rotating about an axis aligned with gravity then otolith signals should not modulate and so the conflict is resolved. The extent of the first mechanism, known as “dumping,” can be quantified with a “dumping index,” which is the difference between the VOR time constants when the head remains upright after it is stopped (e.g., normal post-rotatory nystagmus) and when it is tilted after it is stopped, divided by the head-upright time constant. If tilting the head has no effect on the VOR time constant, then the upright and tilted time constants are the same and dumping index is zero. In contrast, if tilting the head completely suppresses the nystagmus (time constant is zero), then the dumping index is unity. The shift in the eyes rotational axis is assessed as shown for centrifugation (see Figure 2A); if the axis shift is as large as the head tilt (45° in our experiment), then the conflict is also completely resolved.

**Figure 3:**
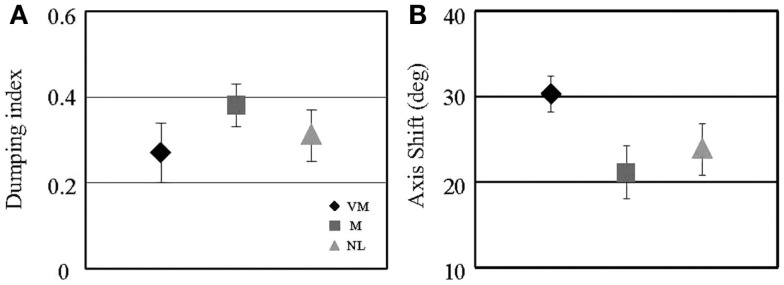
**Characteristics of the eye movement responses in the post-rotational tilt (“dumping”) paradigm in the three subject groups are shown**. Icons are means, and error bars are 1 SE. **(A)** shows the dumping index, as defined in the text, and **(B)** shows the axis shift of the vestibulo-ocular reflex. Subject group sizes range from four to six.

The results of our preliminary study are summarized in Figure 3. We found two differences in the VM group compared to controls using this testing paradigm. Although the extent of post-rotational suppression of the VOR as quantified with the dumping index was the same in the VM, MC, and N groups (Figure 3A; *p* > 0.05 for all comparisons), the amplitude of the axis shift was significantly larger in the VM group compared to the controls (Figure 3B; *p* = 0.01 for VM compared to MC, *p* = 0.03 for VM compared to normal). The meaning of this finding is uncertain, but perhaps it indicates that the VM subjects overestimated the amplitude of the roll head tilt that was produced after the rotational chair decelerated to a stop. This interpretation is consistent with the concept that co-planar canal and otolith cues (which occur in the roll plane during the head tilt) may be integrated in an enhanced manner in VM subjects relative to controls. The second finding was that in the MC and N subjects, the amount of VOR tilt suppression (dumping index) and the size of the VOR axis shift were inversely correlated – in subjects with larger axis shifts, the suppression of the VOR was less (e.g., regression slope of axis shift versus dumping index was −44 for N subjects and −51 for MC subjects, *p* < 0.05 for each). This relationship makes intuitive sense if we consider both of these mechanisms as complementary means to reduce the conflict between the canal and otolith cues. In contrast, in the VM subjects the dumping index and axis shift were uncorrelated (regression slope of +5, *p* = 0.4), indicating that the interaction between the two mechanisms that discharge the intra-vestibular sensory conflict was absent in VM. Since sensory conflict is considered the principal mechanism underlying motion sickness (6) and subjects with VM have greater motion sickness susceptibility than M or N groups (4), one could interpret these findings as a possible explanation for the increased motion sensitivity in VM relative to the control groups.

## Discussion/Hypotheses

The results described above, while preliminary and lacking certain control experiments, provide a confluence of observations suggesting that when activity in canal and otolith organs is modulated in tandem, a pattern of behavioral (perceptual, VOR) abnormalities is present in VM subjects when compared to migraine and normal controls. Before considering the implications of these findings further, it is important to review some issues that potentially complicate the interpretation of the results. While the VOR is a relatively straightforward behavioral correlate of the peripheral vestibular input, percepts of head motion and orientation are clearly dependent on both vestibular and *extra-vestibular* sensory cues. Like prior studies [e.g., Ref. (21)], we used a direction recognition task in the threshold experiment to minimize the influence of vibration and other mechanical cues, we eliminated vision, and minimized audition. Tactile cues are the most challenging to control, as it is possible that perceptual thresholds during roll tilt could be decreased in VM if somatosensory cues were hypersensitive. This is probably not the explanation for our results because: increased tactile sensitivity could not explain the slower development of tilt percepts in the centrifugation paradigm, but rather should produce the opposite effect; the VM subjects differed from the MC control subjects on both perceptual studies; and the MC subjects developed tilt percepts during centrifugation at the same rate as normal controls. None of these observations are consistent with a primarily non-vestibular mechanism that differentiates VM subjects from MC and N controls. Other issues, which require further study but do not appear to be adequate, explanations for our results in these three studies are effects related to habituation (22), motion sickness susceptibility (4), and gender (21).

Given that our results are most consistent with an abnormal vestibular mechanism in VM subjects, the next question is to consider what this abnormality may be at the systems, anatomic, and neurochemical levels. At this point, we clearly are moving beyond our experimental results and into the realm of hypothesis and theory.

At a *systems* level, the results of all three experiments are most consistent with an abnormality in the brain’s *synthesis of canal and otolith* information in patients with VM. The first two experiments suggest some types of increased sensitivity to these dual vestibular inputs, as perceptual thresholds were decreased when the canal and otolith cues were co-planar (during roll tilt) but percepts of tilt developed more slowly when the cues were in conflict (during centrifugation). The third study suggests that the way the brain normally resolves conflicts between canal and otolith signals is not regulated normally in VM, as the relationship between the two mechanisms that serve to minimize this conflict in the post-rotational tilt paradigm (attenuation of the angular velocity signal as evidenced by the horizontal VOR, shift in the estimated axis of head rotation as evidenced by the VOR’s axis shift) lacked the normal pattern in VM.

This hypothesis does not exclude the possibility that other mechanisms also contribute to vestibular symptomatology in patients with VM, and indeed, given the complexity of the potential interactions between migraine and the vestibular system in the brain [reviewed in Ref. (23)], multiple mechanisms are highly probable. Furthermore, our results do not necessarily imply that abnormalities in canal–otolith integration in VM even contribute to vestibular symptomatology, as it is possible, but unlikely that our results are either epiphenomena without symptomatic consequence, or that they are due to repeated vestibular episodes, rather than responsible for them. Given the very high frequency of positional dizziness and nystagmus in VM, however, it seems improbable that patterns of vestibular information produced by the changes in head orientation during these provoking movements play no role in the resultant symptomatology. In addition, we have begun to test perceptual thresholds in patients with Meniere’s disease who have recurrent episodes of vertigo but normal peripheral vestibular function (as assessed by physical examination, caloric and rotational tests, and vestibular evoked potentials). These patients did not demonstrate reductions in perceptual thresholds for any motion paradigm, including roll tilt, which supports our hypothesis that the changes in VM contribute to, rather than result from, repeated episodes of vestibular symptoms.

Based on the hypothesis that canal–otolith integration in the brain is abnormal in VM, the next step is to consider the possible *anatomic* substrate for these changes. Although primary canal and otolith afferents terminate in regions of the vestibular nuclei that are largely segregated, both inputs project to the caudal vermis of the cerebellum (nodulus/uvula), and there is considerable evidence that the synthesis of canal and otolith cues occurs in this region of the brain (12). Purkinje cells in the cortex of the nodulus/uvula inhibit the vestibular nuclei, which are embedded in the velocity storage network via their commissural connections (24). The vestibular nuclei project to the thalamus, which projects in turn to the vestibular regions of the cerebral cortex. While all Purkinje cells in the nodulus/uvula carry integrated canal and otolith signals, only a subset of neurons in the vestibular nuclei and thalamus carry these synthesized motion cues (25). Altered signal processing in the *caudal cerebellar vermis* is therefore an attractive way to explain changes in canal–otolith integration without apparent changes in canal or otolith-mediated responses. Given this possible mechanism, it is interesting to note that minor cerebellar abnormalities related to eye (26) and arm movements (27) have been described in asymptomatic migraine patients, and MRI studies in migraine have noted a propensity for white matter lesions in the cerebellum [(28), although the brain regions that are most typically abnormal on MRI in migraine subjects remains controversial, see for example, Ref. (29)].

If the functional abnormalities responsible for aberrant canal–otolith integration are localized to the cerebellum, what *biochemical* changes associated with migraine could potentially be responsible for these effects? A possible biochemical candidate is calcitonin gene-related peptide (CGRP), a substance that is critical for migraine pathogenesis (30), and is widely distributed throughout the vestibular system. CGRP levels increase during migraine but remain elevated between headaches (31). This neuro-peptide has been identified in the mossy fiber input to the cerebellar nodulus (32), the vestibular nuclei (33), and vestibular efferents (34). While excessive CGRP in each of these locations could presumably result in vertigo, dysfunction in the nodulus would be the most likely cause of enhanced sensitivity to combined canal and otolith stimulation because of its specific role in the synthesis of these vestibular cues. In particular, CGRP inhibits mossy fiber activity in the nodulus (35), so elevated CGRP levels between episodes of vertigo could reduce Purkinje cell activity, thereby disinhibiting the neurons in the vestibular nuclei that receive their projections and contribute to the velocity storage network. The net effect could be amplification of the motion signal detected by the brain during combined canal and otolith activation. This mechanism could also explain the slight increase in VOR time constant in VM patients reported in a recent study (4), although this was not observed in our smaller patient population. Since velocity storage has been linked to the synthesis of canal and otolith cues (25) and motion sickness (36), aberrant control of this central integrator could contribute to both the vertigo and motion intolerance that is characteristic of VM.

Other potential mechanisms could also be postulated to explain some of our experimental findings. For example, for the changes in perceptual thresholds: (a) there are direct connections between brainstem nuclei that are activated during migraine such as the locus coeruleus (LC) and the vestibular nuclei. Levels of tonic and phasic activity in the LC appear related to optimizing task performance (37) and increased LC activity in migraine (38) could therefore result in improved task performance and lower response thresholds. More specifically, roll tilt thresholds at mid-frequencies in normal subjects are lower than those predicted from an optimal linear combination of canal and otolith cues (14), and increased LC activity in VM could optimize this non-linear component of motion detection normally derived from canal–otolith interaction, thereby amplifying the motion cue and lowering perceptual thresholds; (b) the release of inflammatory agents sensitizes primary trigeminal afferents, their target neurons in the trigeminal nucleus (39), and thalamic neurons that receive dual input from the trigeminal nucleus and other sensory systems (40). Since there are direct projections from the trigeminal nucleus to the vestibular nuclei, sensitized neurons in the trigeminal nucleus could potentially enhance the sensitivity of neurons in the vestibular nuclei. A similar hypothesis could be applied to thalamic neurons if they were sensitized by dual innervation from the vestibular and trigeminal nuclei. If this were the underlying mechanism for our results, the sensitized trigeminal neurons should primarily influence the subset of vestibular neurons in the vestibular nuclei or thalamus that carry the integrated canal–otolith signal; and (c) proprioceptive afference from the neck muscles could potentially affect the post-rotational tilt experiment, where the head was moved relative to the body (the head and body did not move relative to each other in the roll tilt threshold or the centrifugation experiments). Since the brain uses vestibular, visual, efferent, and proprioceptive information to encode head motion, if VM subjects have aberrant proprioceptive signals this could possibly affect how the brain uses vestibular information to control eye movements. It seems more likely, however, that cervical proprioception would primarily influence percepts of head motion and orientation, and in this manner could produce misperceptions of head motion (or “dizziness”) in VM subjects during normal activities.

Whichever of these mechanisms were aberrant in VM, they could contribute to the baseline increase in motion sickness sensitivity that is characteristic of migraine and particularly VM (4). If the perceptual abnormality were further enhanced by an ictal increase in CGRP release or change in LC activity, for example, patients may overestimate the amplitude of head movements and this could generate abnormal illusions of motion, particularly when the head is reoriented relative to gravity.

*Future directions* for our work are to complete the three experiments described above, which are currently in preliminary form. Following that, we plan to expand these types of behavioral studies to include *visual–vestibular* interactions, the effect of migraine *therapy* on these findings, and *functional imaging*. Regarding vision, patients with migraine have problems distinguishing between visual signal and noise (41), which is the basis of threshold testing, and it would be interesting to examine how visual and vestibular signal and noise could potentially interact. Regarding migraine therapy, while the effects of all abortive and prophylactic medications would be of interest, the possible mechanism outlined above makes testing the effects of CGRP receptor antagonists (42) or antibodies directed against CGRP or its receptors (43) on vestibular-mediated behavioral responses of particular interest. Regarding imaging, we have performed fMRI on one subject to date using a hypercapnic (breath-holding) approach to accentuate changes in blood flow. Unlike the recently published study (19), which used caloric stimuli, we did not use an external stimulus other than the MRI magnet, which has been recently demonstrated to activate the peripheral vestibular organs (44). In the supine orientation in the scanner, activation of the lateral canals by the magnet (which has been evidenced by the brisk horizontal nystagmus induced in the scanner) would produce a canal–otolith conflict, as the canals would sense yaw rotation about an earth-horizontal axis, well the otoliths would not modulate in the manner that would normally be produced by this form of motion. Interestingly, we found extensive changes in both the lateral cerebellar hemispheres and the cerebellar vermis in our VM subject (compared to migraine and normal subjects scanned in the same manner), results, which differ from the thalamic changes found during caloric stimulation (19).

In conclusion, given the multiple potential interactions between migraine and the vestibular system, it is likely that vertigo in VM is multi-factorial and could include labyrinthine as well as neurologic components. Despite this complexity, our preliminary studies suggest that there is a pattern of abnormalities in VM subjects that could be explainable by changes in the central integration of canal and otolith signals. These findings suggest a possible cerebellar anatomic substrate for vestibular dysfunction in VM, and this localization focuses attention on possible biochemical/neurotransmitter changes that may be present both during and between vertigo episodes in these patients. Furthermore, since our testing approach may be able to differentiate VM from both migraine and normal subjects, this may eventually prove to be a fruitful pathway to develop a diagnostic test for VM, a disorder that currently lacks a pathognomonic finding.

## Author Contributions

Susan King, Joanne Wang, Adrian J. Priesol, and Richard F. Lewis performed experiments, analyzed data, and wrote the manuscript.

## Conflict of Interest Statement

The authors declare that the research was conducted in the absence of any commercial or financial relationships that could be construed as a potential conflict of interest.
